# Catastrophic health expenditure incidence and its equity in China: a study on the initial implementation of the medical insurance integration system

**DOI:** 10.1186/s12889-019-8121-2

**Published:** 2019-12-30

**Authors:** Huan Liu, Hong Zhu, Jiahui Wang, Xinye Qi, Miaomiao Zhao, Linghan Shan, Lijun Gao, Zheng Kang, Mingli Jiao, Lin Pan, Ruohui Chen, Baohua Liu, Qunhong Wu, Ning Ning

**Affiliations:** Department of Social Medicine, Health Management College, Harbin Medical University, Harbin, China

**Keywords:** Catastrophic health expenditure, Medical insurance integration system, Equity, Influencing factors

## Abstract

**Background:**

By 2013, several regions in China had introduced health insurance integration policies. However, few studies addressed the impact of medical insurance integration in China. This study investigates the catastrophic health expenditure and equity in the incidence of catastrophic health expenditure by addressing its potential determinants in both integrated and non-integrated areas in China in 2013.

**Methods:**

The primary data are drawn from the fifth China National Health Services Survey in 2013. The final sample comprises 19,788 households (38.4%) from integrated areas and 31,797 households (61.6%) from non-integrated areas. A probit model is employed to decompose inequality in the incidence of catastrophic health expenditure in line with the methodology used for decomposing the concentration index.

**Results:**

The incidence of catastrophic health expenditure in integrated areas is higher than in non-integrated areas (13.87% vs. 13.68%, respectively). The concentration index in integrated areas and non-integrated areas is − 0.071 and − 0.073, respectively. Average household out-of-pocket health expenditure and average capacity to pay in integrated areas are higher than those in non-integrated areas. However, households in integrated areas have lower share of out-of-pocket expenditures in the capacity to pay than households in non-integrated areas. The majority of the observed inequalities in catastrophic health expenditure can be explained by differences in the health insurance and householders’ educational attainment both in integrated areas and non-integrated areas.

**Conclusions:**

The medical insurance integration system in China is still at the exploratory stage; hence, its effects are of limited significance, even though the positive impact of this system on low-income residents is confirmed. Moreover, catastrophic health expenditure is associated with pro-poor inequality. Medical insurance, urban-rural disparities, the elderly population, and use of health services significantly affect the equity of catastrophic health expenditure incidence and are key issues in the implementation of future insurance integration policies.

## Background

Generally, catastrophic health expenditure (CHE) represents out-of-pocket (OOP) payments for health care exceeds a specified threshold of household’s income or household’s capacity to pay (CTP) [1–3]. There is no consensus on the threshold above which health expenditures are considered catastrophic. For example, OA et al. defined CHE as direct OOP medical costs exceeding 10% of the monthly household income [2]. The World Health Organization (WHO) defined financial catastrophe as the OOP expenditure exceeding 40% of the household income net of subsistence needs [3].

Surveys in 89 countries, covering 89% of the world’s population, suggested that 150 million people globally suffer financial catastrophes every year due to OOP medical costs [4]. Developed countries have advanced medical insurance and medical service systems that protect households from catastrophic spending. Choi et al. found that only 3.5% of Korean households suffered from CHE in 2008 (according to the 40% threshold) [5]. However, the incidence of CHE (H_cat_) in developing countries is relatively high, especially in low-income developing countries. Ghimire et al. utilized nationally representative data for Nepal and found that the cumulative H_cat_ is 10.3% per month [6]. A clustered sample survey conducted in Iran in 2008 showed that 11.8% of households faced CHE [7]. In Georgia, the results of a survey showed that 19% of households seeking care had to borrow money or sell personal items to pay for health care, and 16% were unable to afford the medications prescribed [8]. According to a cross-sectional study, in 2008, about 13% of families experienced CHE in China [9].

Previous studies found that several factors are associated with CHE: household size, presence of family members aged over 65 or less than five years, household members with a chronic disease, residence, hospitalized family members, income, insurance, gender of the household head, and education level, among the others, have been significantly associated with CHE [6, 10–12]. Falconi indicated that poorer, rural, and smaller households, as well as households with older members and individuals with chronic conditions, have larger odds of facing CHE [13]. However, only a few studies have addressed inequality in CHE. Boing et al. found that the poorest households and households headed by the least-educated individuals significantly contribute to increasing social inequality [14]. Moradi et al. showed that income is the most significant determinant of inequality in facing CHE [15]. Wang et al. used a nationally representative dataset and found that household size, per capita income, family members above 65 years of age, and family members with two or more chronic diseases significantly increase CHE inequality [16].

At the end of 1998, China launched a government-run mandatory insurance program—the urban employee basic medical insurance (UEBMI)—to replace the previous labor medical insurance [17, 18]. The UEBMI covered urban employees (including retired and rural-to-urban migrant workers) with funds contributed by employers and employees [19], the fund was divided into two parts with individual accounts set mainly to cover outpatients service or buy drugs, while the major fund was pooled together mainly cover the inpatients expenditure. In 2003, the new rural cooperative medical system (NRCMS) was launched as a voluntary insurance program to reduce the financial burdens of high medical expenses for uninsured people with rural household registration, including rural-to-urban migrant workers and regular rural residents [20]. Its funding comes from individual contributions, collective support, and government funding. Four years later, the urban residents’ basic medical insurance (URBMI) was developed targeting the elderly, students, children, self-employed, and unemployed urban residents not covered by the UEBMI and NRCMS schemes [21]. Its funding sources were based on the payments of individual urban residents, supplemented by government subsidies. The three schemes are administered by different agencies. NRCMS is administered by the Ministry of Health, whereas URBMI and UEBMI are both administered by the Chinese Ministry of Human Resources and Social Security. In addition, the pool levels for the three schemes are different. NRCMS pools its funds at the county level in rural areas, while UEBMI and URBMI pool their funds at the municipal level in urban areas [22]. In 2010, the Chinese government acknowledged these three categories—UEBMI, URBMI, and NRCMS—of the basic medical insurance system [23]. These insurance schemes have enabled China to achieve near-universal health coverage, with more than 1.3 billion Chinese people (about 97% of the population) having some form of medical insurance [24].

The urban-rural dual structure of the current social health insurance in China is designed for ease of administration [25]. However, significant urban-rural disparities in health care and social equality have negatively affected the progress toward universal health care. A study on patients suffering from chronic disease in rural China indicated that the NRCMS offers only a limited degree of financial protection [26]. Some scholars also found that the impact of NRCMS is of little help to adequately protect the insureds from CHE or poverty caused by diseases, especially among the poor [27–29]. Further, the large differences in insurance coverage [30] and health care benefits [31] among the three basic medical insurance systems are the crucial cause of the significant inequalities in financial protection. Yan et al. found that the H_cat_ among URBMI and NRCMS enrollees is significantly higher than that among UEBMI enrollees, and that for NRCMS is the highest [32]. Other fragmented problems also need to be solved in the three separated medical insurance system, such as repetition of multiple insurance coverages [18], restriction of the “floating population” [25, 33], and inefficient management [34]. Hence, it is necessary to establish a proper transferring mechanism to integrate UEBMI, URBMI, and NRCMS to solve these fragmented problems.

In 2009, the Chinese government assessed the need to build an urban-rural integrated security management system [35]. Some regions attempted to integrate URBMI and NRCMS into the urban-rural resident basic medical insurance (URRBMI) [36]. The municipalities of five provincial administrative regions (Tianjin and Chongqing, Qinghai province, Ningxia Hui autonomous region, and Xinjiang Production and Construction Corps), 41 prefecture cities, and 162 counties (districts and county-level cities) had already established the URRBMI at the end of 2011 [37]. In addition, some advanced regions tried to integrate the three basic medical insurance schemes into a universal social basic medical insurance (USBMI).

Accordingly, this study’s contribution to the literature is twofold. First, although a medical insurance integration system has been implemented in some areas of China, only a few studies have evaluated the impact of its implementation. Second, previous studies have identified several potential determinants of equity in the H_cat_. However, the existence of factors that can systematically hinder equity after the implementation of the medical insurance integration system needs to be explored. Thus, this study investigates the impact of the current medical integration system on reducing the H_cat_ and improving equity in H_cat_ in China while exploring the determinants of inequality in H_cat_.

## Methods

### Data sources

The primary data used in this study are drawn from the fifth National Health Services Survey (NHSS, 2013) conducted by the Center for Health Statistics and Information of the Ministry of Health of China. The survey has been carried out every five years since 1993 and collects information regarding the general status of the family, personal status of family members, illness of family members in the two weeks before the survey, hospitalization within one year before the survey, and situation of children under five and women aged 15–64.

### Sampling method

The fifth NHSS adopts four-stage, stratified, random sampling, involving 31 administrative divisions in China. A total of 156 cities are randomly selected in the first stage, representing six geographical locations (eastern cities, eastern rural areas, central cities, central rural areas, western cities, and western rural areas). In every city, five sampled townships are randomly selected in the next stage, for a total of 780 townships. Among these, 1560 villages are selected in the next stage. Finally, 273,688 respondents (from 93,613 families) are investigated. The response rate is 82.1% (at the individual level).

In this study, to assess the determinants of inequality in the H_cat_ in the pioneer integrated areas (IAs) of China, we address pilots that implemented the local medical insurance integration policy before the fifth NHSS. The sample comprises areas that implemented the integration (integrated pilots). As a result, the sample comprises 32 integrated pilots of 156 sample areas, which belong to 13 administrative divisions. In three regions (Tianjin, Chongqing, and Ningxia), every sample area represents an integrated pilot, and comparable regions (Beijing, Shanxi, and Hebei) per capita gross domestic product and consumer price index (as of 2012) are used for the analysis. All sample areas of the comparable regions are referenced as non-integrated pilots. In the other 10 regions, some sample areas are integrated pilots, and the remaining sample areas are referenced as non-integrated pilots. Finally, 13 regions (32 integrated pilots) are identified as “IAs,” and the corresponding 13 regions (53 non-integrated pilots) are identified as “non-integrated areas” (NIAs) (see Additional file 1: Table S1).

After the data cleaning process, the final sample comprises 19,788 households (38.4%) in IAs and 31,797 households (61.6%) in NIAs. Quality control has been implemented by supervisors charged with guiding and inspecting every step of the survey. Face-to-face household interviews have been conducted by qualified investigators, and 5% of the sample families have been revisited to check the accuracy of the data (above 95%; otherwise, return visits were made to reinvestigate).

### Variables

The relevant characteristics of the head of the household include gender (men and women), marital status (married or other), educational attainment (illiterate, primary school, junior high school, high school, and above), occupation (employed, retired, and other), and medical insurance (UEBMI, URBMI, NRCMS, USBMI, URRBMI, mix medical insurance, uninsured, and other). The relevant household characteristics include household location (eastern, middle, and western areas), residence (urban and rural), household size (≤2, 3–4, ≥5), and preferred medical institution (primary and non-primary). Household income is ranked in five quintiles according to the WHO, and OOP health expenditure, food consumption expenditure, and medical consumption expenditure are accounted for at the household level. Some household variables are measured as “yes” or “no,” based on the following questions: “Has any household member been hospitalized in the past year?” “Is there anyone with a chronic disease in the family?” “Is there anyone over 60 years old in the family?” “Is there anyone under five years old in the family?”

### Statistical analysis

In this study, CHE is analyzed based on measures recommended by Wagstaff et al., which include the H_cat_, CHE gap, CHE inequality, and decomposition of CHE inequality [38]. In this study, the 40% threshold recommended by the WHO is used to calculate CHE [3]. Monthly household consumption expenditure is ranked into quintiles after adjusting it for standard household size. This adjustment allows any differences in health spending across countries to be attributed to factors other than the differential composition of their populations. The poverty line is defined by subsistence spending as the average monthly food expenditure of the household whose food expenditure as a share of total household consumption expenditure falls between the 45th and 55th percentiles of the sample. The subsistence spending of each household is calculated as the poverty line multiplied by the standard household size. If a household’s total expenditure is less than this figure, the household is categorized as poor [10]. Household non-subsistence spending is used as a proxy for CTP. However, whenever food expenditure is less than subsistence spending, CTP is defined as total expenditure minus food expenditure.

H_cat_ describes the proportion of households facing CHE in the sample. In addition to a catastrophic payment headcount, this study adopts a measure analogous to the poverty gap, called “CHE gap.” The mean CHE gap (G_cat_) and mean positive CHE gap (MPG_cat_) are used to measure the intensity and severity of CHE, respectively.

The concentration index (CI) is employed to measure the extent of economic inequality in the H_cat_. CI is defined as twice the area between the concentration curve and the line of equality, and it lies between [− 1,1] [39]. Its positive value indicates that inequality in the H_cat_ is more concentrated among the rich, while a value equal to zero indicates that there is no inequality. The larger the absolute value of CI, the greater the H_cat_. In line with Wagstaff et al., the WE Cat index is calculated to modify the catastrophic payment headcount by the individual rank in the income distribution (which corresponds to the weighted H_cat_) [38]..

Inequality can be further explained by decomposing the CI into its determinants. Various decomposition methods can quantify each determinant’s contribution to measuring economic inequality while controlling for other determinants. A probit model is employed to decompose the inequality of the H_cat_ in line with the decomposition method used for the CI. All analyses are performed in Stata version 11.0, and *p* < .05 is the threshold for statistical significance.

## Results

### Basic features of sample families

Table 1 reports the summary statistics of the households and the household heads’ characteristics. Both in IAs and NIAs, household heads are predominantly men, married, with junior high school education, and employed. The primary insurance scheme for the heads of household is NRCMS (56.2%) in NIAs and URRBMI (56.1%) in IAs. Households in IAs are mainly concentrated in the eastern urban areas. Compared with NIAs, households in IAs are more likely to attend non-primary medical institutions (23.2% vs .18.8%).

**Table 1:** Characteristics of households and household heads

Variable	Total *N* (%)	Integrated area *n* (%)	Non-integrated area *n* (%)
Household head			
Gender			
Men	38,221 (74.1)	14,706 (74.3)	23,515 (74.0)
Women	13,364 (25.9)	5082 (25.7)	8282 (26.0)
Marital status			
Unmarried and other	8201 (15.9)	3339 (16.9)	4862 (15.3)
Married	43,384 (84.1)	16,449 (83.1)	26,935 (84.7)
Educational attainment			
Illiterate	5332 (10.3)	2052 (10.4)	3280 (10.3)
Primary school	14,594 (28.3)	5553 (28.1)	9041 (28.4)
Junior high school	18,050 (35.0)	6740 (34.1)	11,310 (35.6)
High school and above	13,609 (26.4)	5443 (27.5)	8166 (25.7)
Occupation			
Employed	35,080 (68.0)	13,074 (66.1)	22,006 (69.2)
Retired	9720 (18.8)	3844 (19.4)	5876 (18.5)
Other	6785 (13.2)	2870 (14.5)	3915 (12.3)
Medical insurance			
UEBMI	14,697 (28.5)	6171 (31.2)	8526 (26.8)
URBMI	2715 (5.3)	–	2715 (8.5)
NRCMS	17,875 (34.7)	–	17,875 (56.2)
USBMI	930 (1.8)	930 (4.7)	–
URRBMI	11,104 (21.5)	11,104 (56.1)	–
Mixed medical insurance	3050 (5.9)	1106 (5.6)	1944 (6.1)
Uninsured and other	1214 (2.4)	477 (2.4)	737 (2.3)
Household			
Location			
Eastern	20,401 (39.5)	10,791 (54.5)	9610 (30.2)
Middle	14,384 (27.9)	2397 (12.1)	11,987 (37.7)
Western	16,800 (32.6)	6600 (33.4)	10,200 (32.1)
Residence			
Urban	26,382 (51.5)	10,801 (54.6)	15,581 (49.0)
Rural	25,203 (48.9)	8987 (45.4)	16,216 (51.0)
Household size			
≤ 2	23,459 (45.5)	8882 (44.9)	14,577 (45.8)
3–4	21,351 (41.4)	8077 (40.8)	13,274 (41.7)
≥ 5	6775 (13.1)	2829 (14.3)	3946 (12.4)
Hospitalized member			
Yes	11,147 (21.6)	3987 (20.1)	7160 (22.5)
No	40,438 (78.4)	15,801 (79.9)	24,637 (77.5)
Member with chronic disease			
Yes	23,634 (45.8)	9342 (47.2)	14,292 (44.9)
No	27,951 (54.2)	10,446 (52.8)	17,505 (55.1)
Member > 60 years of age			
Yes	23,367 (45.3)	9030 (45.6)	14,337 (45.1)
No	28,218 (54.7)	10,758 (54.4)	17,460 (54.9)
Member < 5 years of age			
Yes	8712 (16.9)	3210 (16.2)	5502 (17.3)
No	42,873 (83.1)	16,578 (83.8)	26,295 (82.7)
Preferred medical institution			
Primary medical institutions	41,028 (79.5)	15,193 (76.8)	25,835 (81.2)
Non-primary medical institutions	10,557 (20.5)	4595 (23.2)	5962 (18.8)
Household income			
Quintile I (Poorest)	10,328 (20.0)	3962 (20.0)	6366 (20.0)
Quintile II	10,329 (20.0)	3954 (20.0)	6375 (20.0)
Quintile III	10,298 (20.0)	3957 (20.0)	6341 (19.9)
Quintile IV	10,345 (20.1)	3958 (20.0)	6387 (20.1)
Quintile V (Richest)	10,285 (19.9)	3957 (20.0)	6328 (19.9)

*UEBMI* urban employee basic medical insurance, *URBMI* urban resident basic medical insurance, *NRCMS* new rural cooperative medical scheme, *URRBMI* urban and rural residents’ basic medical insurance system, *USBMI* universal social basic medical insurance

### Catastrophic health expenditure

At a 40% threshold, the poorest households face the highest proportion of CHE occurrence compared with other quintiles, both in IAs and NIAs; the CI is negative both in IAs and NIAs, thus suggesting that inequality in CHE is biased toward low-income people. The absolute value of the CI is slightly lower for IA households than NIA households. Furthermore, the WE Cat of IA households is slightly higher than that of NIA households (Table 2).

**Table 2:** H_cat_, CHE gap, CI, and WE Cat at the 40% threshold

	Integrated areas	Non-integrated areas
H_cat_ (%)		
Quintile I (Poorest)	17.19	17.45
Quintile II	15.33	13.95
Quintile III	12.82	12.46
Quintile IV	11.99	11.55
Quintile V (Richest)	11.98	12.99
Total	13.87	13.68
G_cat_ (%)	2.67	2.61
MPG_cat_ (%)	19.25	19.08
CI	−0.071	−0.073
WE Cat (%)	14.85	14.68

*H*_*cat*_ catastrophic health expenditure incidence, *G*_*cat*_ the mean catastrophic health expenditure gap, *MPG*_*cat*_ the mean positive catastrophic health expenditure gap, *CI* concentration index, *WE Cat* the weighted catastrophic health expenditure incidence

Table 3 presents the average household OOP health expenditure, average household CTP, and proportion of households affected by poverty and medical impoverishment. In IAs, addressing the income-based groups from the lowest to highest income, the average OOP expenditure ranges from 1041.68 yuan up to 8445.06 yuan, and the OOP share in CTP ranges from 21.90% down to 14.90%. In NIAs, addressing the income-based groups from the lowest to highest income, the average OOP expenditure ranges from 1023.66 yuan up to 8881.48 yuan, and the OOP share in CTP ranges from 21.86% down to 16.08%. Overall, the average household OOP health expenditure and average household CTP in IAs are higher than those in NIAs. However, households in IAs face a lower OOP share in CTP than households in NIAs.

**Table 3:** Summary statistics for OOP, CTP, OOP/CTP**,** and medical impoverishment

	Integrated areas					Non-integrated areas				
Household income	Quintile I	Quintile II	Quintile III	Quintile IV	Quintile V	Quintile I	Quintile II	Quintile III	Quintile IV	Quintile V
OOP (Yuan)	1144.72	2225.46	3141.78	4415.60	9454.39	974.18	1934.9	2743.83	3945.66	8255.18
Average CTP (Yuan)	5637.43	12,319.50	19,411.68	29,691.08	65,956.65	4794.29	10,302.79	16,255.69	24,766.32	50,333.34
OOP/CTP (%)	21.60	19.06	17.11	15.74	14.51	22.09	19.65	17.56	16.37	15.83
Medical impoverishment (%)	6.46	13.10	5.56	1.82	1.77	7.60	15.40	5.25	1.96	1.30

*OOP* out-of-pocket, *CTP* capacity to pay

### Illness behavior and utilization in IAs

**Fig. 1:**
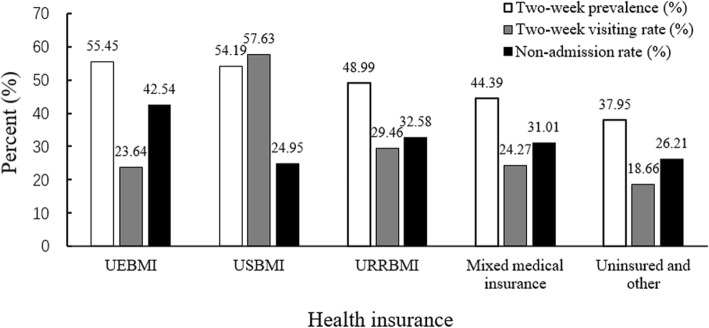
Illness behavior and utilization in integrated areas. This figure explains the two-week prevalence, two-week visiting rate, and non-admission rate among different medical insurance enrollees in integrated areas. “Non-admission rate” refers to the percentage of patients needing treatment who are not treated in the two weeks before the survey

“Non-admission rate” is defined as the percentage of patients needing treatment who are not treated in the two weeks before the survey. In IAs, households for which the household head is enrolled in the UEBMI have the highest prevalence rate and non-admission rate in the two weeks before the survey (Fig. 1).

### Decomposition of inequality of CHE

Table 4 reports the CI and the relative contributions of each determinant of the H_cat_ inequality in both IAs and NIAs. The β_k_ coefficient indicates that hospitalized household members, household population aged two or less, family members with chronic diseases, household population equal to three or four members, family members over 60 years of age, residence in rural areas, and gender (men) are associated with an increase in the probability of risk for incurring CHE, both in IAs and NIAs. The URRBMI has a positive impact on the CHE occurrence rate among household in IAs.

**Table 4:** The disintegration of H_cat_ inequality in integrated areas and non-integrated areas

Integrated areas				Non-integrated areas			
Variable	β	CI	Contribution (%)	Variable	β	CI	Contribution (%)
Hospitalized member ^***^			−42.48	Hospitalized member^***^			−44.00
Yes	0.946	0.096		Yes	0.881	0.099	
No	Reference			No	Reference		
Chronic disease member ^***^			−4.85	Chronic disease member^***^			−9.64
Yes	0.396	0.015		Yes	0.434	0.029	
No	Reference			No	Reference		
Member > 60 years of age ^***^			19.32	Member > 60 years of age ^***^			12.99
Yes	0.259	−0.098		Yes	0.233	− 0.073	
No	Reference			No	Reference		
Member < 5 years of age		− 0.014	0.15	Member < 5 years of age			− 0.60
Yes	0.039			Yes	−0.042	− 0.050	
No	Reference			No	Reference		
Preferred medical institution			3.46	Preferred medical institution ***			−12.23
Primary medical institutions	0.040	−0.070		Primary medical institutions	−0.113	− 0.076	
Non-primary medical institutions	Reference			Non-primary medical institutions	Reference		
Household income			13.38	Household income			23.83
Quintile I (Poorest)	Reference			Quintile I (Poorest)	Reference		
Quintile II	0.032	−0.400		Quintile II	−0.037	− 0.399	
Quintile III	−0.035	0.000		Quintile III ^**^	−0.088	0.001	
Quintile IV	−0.067	0.400		Quintile IV ^**^	−0.113	0.401	
Quintile V (Richest)	−0.002	0.800		Quintile V (Richest)	− 0.054	0.801	
Household size			7.01	Household size			1.57
≤ 2 ^***^	0.788	− 0.026		≤ 2 ^***^	0.646	−0.019	
3–4 ^***^	0.347	0.038		3–4 ^***^	0.294	0.039	
≥ 5	Reference			≥ 5	Reference		
Sex			1.22	Sex			2.70
Men ^***^	0.095	−0.012		Men ^***^	0.120	−0.019	
Women	Reference			Women	Reference		
Marital status			2.34	Marital status			3.90
Unmarried and other ^**^	−0.066	0.025		Unmarried and other ^**^	−0.091	0.029	
Married	Reference			Married	Reference		
Educational attainment			45.47	Educational attainment			33.41
Illiterate	Reference			Illiterate	Reference		
Primary school ^***^	−0.180	−0.181		Primary school ^***^	−0.213	− 0.183	
Junior high school ^***^	−0.346	−0.003		Junior high school ^***^	−0.321	0.007	
High school and above ^***^	−0.486	0.317		High school and above ^***^	−0.401	0.327	
Occupation			9.16	Occupation			−7.06
Employed ^***^	−0.367	0.006		Employed ^***^	−0.394	−0.029	
Retired ^**^	−0.139	0.156		Retired ^*^	−0.091	0.283	
Other	Reference			Other	Reference		
Location of residences			−0.39	Location of residences			4.30
Eastern ^***^	0.045	0.045		Eastern ^***^	−0.109	0.080	
Middle ^***^	−0.126	−0.126		Middle ^***^	−0.186	0.000	
Western	Reference			Western	Reference		
Residence			18.55	Residence			23.88
Urban	Reference			Urban	Reference		
Rural ^***^	0.127	−0.194		Rural ^***^	0.148	−0.189	
Medical insurance			29.89	Medical insurance			60.73
UEBMI	0.117	0.232		UEBMI ^***^	−0.262	0.354	
USBMI	−0.185	0.303		URBMI	−0.006	−0.016	
URRBMI ^*^	0.205	−0.191		NRCMS	0.105	−0.195	
Mixed medical insurance	−0.165	0.357		Mixed medical insurance	−0.140	0.252	
Uninsured and other	Reference			Uninsured and other	Reference		

*CI* concentration index, *UEBMI* Urban Employee Basic Medical Insurance, *URBMI* Urban Resident Basic Medical Insurance, *NRCMS* New Rural Cooperative Medical Scheme, *URRBMI* urban and rural residents’ basic medical insurance system, *USBMI* Universal Social Basic Medical Insurance

* *p* < 0.05; ** *p* < 0.01; *** *p* < 0.001

The third and seventh columns in Table 4 imply the extent to which the respective variable is distributed across wealth. For example, in IAs, the CIs of some determinants such as men, rural, URRBMI, and midland, are negative, meaning that these features are more concentrated among people of lower economic status. In contrast, mixed medical insurance, UEBMI or USBMI, high school and above education, and employed or retired have a positive CI, thus implying that these features are more concentrated among people of higher economic status.

A positive contribution to socioeconomic inequality means that the considered variable increases inequality. Results in the fifth and last columns of Table 4 show that the majority of observed inequalities in the H_cat_ in IAs can be attributed to educational attainment (45.47%), medical insurance (29.89%), family members over 60 years of age (19.32%), area (18.55%), and household income (13.38%). Hospitalized members (− 42.48%), family members with chronic diseases (− 4.85%), and location of residence (− 0.39%) are negatively related to CHE inequality, thus implying that these factors reduce CHE inequality in IAs. The total percentage contribution is 102.33%, which means that 2.33% of the positive contribution to inequality in the H_cat_ is explained by the error term of the regression. In NIAs, the primary positive contribution to inequality is associated with medical insurance (60.73%), educational attainment (33.41%), area (23.88%), household income (23.83%), and family members over 60 years of age (12.99%). Hospitalized family members (− 44.00%), preferred medical institution (− 12.23%), family members with chronic diseases (− 9.64%), occupation (− 7.06%), and family members below five years old (− 0.60%) are negatively related to CHE inequality in NIAs. The total percentage contribution is 93.77%, which implies that 6.23% of the negative contribution to inequality in the H_cat_ is explained by the error term of the regression.

## Discussion

This study utilized nationally representative data to analyze the incidence, intensity, and inequality of H_cat_ for households in IAs and NIAs after the implementation of the pilot policy of medical insurance integration in China. The results of this study can support decision makers in formulating policies and relieve the economic burden of disease in vulnerable groups.

The CHEs in both IAs and NIAs in China was calculated. The H_cat_ in IAs was higher than that in NIAs. Meanwhile, compared with the results of the fourth NHSS [10], the H_cat_ in IAs has not decreased significantly over the sample period (13% in the fourth NHSS vs. 13.87% in this study). According to the incidence of catastrophic health expenditure, the effect of health insurance integration may not be ideal. However, we cannot ignore the rapid growth in health service demand, and medical expenses in China may have played a significant role. The two-week prevalence increased from 18.86 in 2008 to 24.10 in 2013 [40], and per capita hospitalization cost increased from 5234.1 yuan in 2008 to 7858.9 yuan in 2013 [40, 41]. In addition, the aging of the Chinese population is also worthy of attention. The proportion of the population aged over 65 in China has risen from 8.3% in 2008 to 9.7% in 2013 [42]. Furthermore, with the substantial increase in reimbursement level [43], integrated medical insurance may motivate patients to seek treatment, especially in rural areas [44], which may also influence the H_cat_. Hence, we cannot completely deny the effect of the current medical reform and medical insurance integration policies.

In both IAs and NIAs, the poorest families face the lowest OOP expenditure but experience the highest share of OOP payment for health care. This result confirms the findings of a previous study, which argued that low-income families pay a somewhat higher ratio of OOP expenses relative to their household incomes [45]. OOP expenses are higher in the highest income quintile compared with the lowest income quintile, but households in the highest income quintile suffer a minimal catastrophic impact. This result suggests that although the richest households pay more for health care, they are less likely to suffer a change in their living standards or incur debt due to health care expenses [46]. Furthermore, the proportions of medical impoverishment for poverty and sub-poverty residents in IAs were lower than in NIAs. Compared with results of the fourth NHSS [10], this study’s results show that, for poverty and sub-poverty residents, the proportion of medical impoverishment in IAs has significantly decreased (Quintile I: 10.6% in the fourth NHSS vs. 6.46% in this study; Quintile II: 19.1% in the fourth NHSS vs. 13.10% in this study). Therefore, the positive impact of the medical insurance integration system on low-income residents is confirmed.

In fact, after implementation of the medical insurance integration policy, the number of enrollees, proportion of reimbursement, and overall planning level have continued to increase, and the medical insurance catalogue has expanded [47]. However, according to the data of this study, we can still show that the initial effect of medical insurance integration is not significant. Shan and colleagues found that nearly half of the respondents were dissatisfied with the current medical insurance integration reforms [18]. Some scholars have indicated that the current medical insurance integration has exposed some problems, especially equality issues [19, 48]. Therefore, we need to “apply medicine according to indications” and provide a more effective policy adjustment basis for the next stage of China’s medical insurance integration system.

In this study, CI is used to measure inequality in the H_cat_. The results indicate that CHE is characterized by inequality concentrated among the poor in both IAs and NIAs. After decomposing the inequality in the H_cat_, we found that the main factors causing inequality are very similar in both IAs and NIAs. In other words, these target issues have still not been properly addressed.

Whether in IAs or NIAs, medical insurance is found to significantly contribute to inequality. In IAs, URRBMI is still at the exploratory stage and contributes in favor of the poor. URRBMI adopts the “financing by stages, and linking payment with treatment” strategy to adapt to the consumption capacity of urban and rural residents characterized by different economic levels. However, it also stimulates an invisible inequity, which concentrates on the poor. Low-income residents generally choose the financing level of medical insurance characterized by a low payment threshold and can only benefit from low levels of reimbursement [49]. This phenomenon reflects the heavy medical burden for economically disadvantaged groups, which remains unsolved.

Interestingly, compared with uninsured populations, URRBMI enrollees are positively related to the risk probability of increasing CHE. Several possible explanations exist for this phenomenon. First, URRBMI enrollees have shown a higher prevalence and visiting rate in the two weeks before the survey compared with the uninsured population. In other words, URRBMI enrollees have greater potential to use health services, which, in turn, increases the risk probability of CHE. Second, the prevalence of the untreated among the URRBMI enrollees is also higher than among the uninsured population. The absence of a doctor’s visit may lead to aggravation of the patient’s condition and lead to CHE. Third, the higher CHE risk probability may be caused by adverse selection in the URRBMI policy. The URRBMI shares the patient’s health expenses and reduces the cost burden on families. Thus, people in poor health conditions may be more willing to participate in the URRBMI compared with uninsured people, who are generally healthy.

Significant deficiencies still exist in China’s medical insurance integration policy. In the implementation of future insurance integration policies, the focus should shift to the health needs and payment capacity of all classes of citizens. In addition, a reasonable fundraising and payment mechanism needs to be established to reduce the inequality caused by medical insurance.

This study’s results suggest that residency positively contributes to inequality in H_cat_. In China, due to the dual structure of urban and rural areas, the urban-rural income gap is significant. Previous studies found that the urban-rural income gap accounts for the majority of the national income gap [50, 51]. In addition, the unequal distribution of health resources between urban and rural areas in China further exacerbates the disparities in the health level of urban and rural residents. Wu et al. argued that rural residents are much more financially vulnerable to health crises, and most CHE cases are attributed to rural families [52]. The current medical insurance integration system has only achieved the unified management of urban and rural systems, but gaps still exist between urban and rural residents in their ability to purchase health services. Sun et al. argued that leveling the reimbursement ratios between urban and rural residents is needed for achieving health equality [37]. In fact, rural residents are the most supportive of health care insurance integration, due to the most common reason of achieving equal access to health care services [53]. However, Liu suggested that because the current URRBMI cannot significantly narrow the urban-rural difference in actual compensation rates, it does not have a substantial impact on the level of medical service utilization in China [54]. Therefore, after the integration of the urban and rural medical insurance system, the equality of the financing burden for rural residents should be addressed. In areas with large urban-rural gaps, “one system and two files” or “one system and multiple files” can be implemented, allowing rural residents to choose between various grades, and the transition to “one system and one file” may be pursued when appropriate. In addition, the government needs to invest more funds to further expand the social medical insurance programs for rural low-income people to avoid CHE.

The education level of the household head contributes to pro-poor inequality in the H_cat_. This may be due to the relatively poor health care awareness of the heads of households with lower levels of education, which, in turn, makes them more likely to incur CHE. Provision of fair access to education is an aspect that cannot be ignored in the development of social security systems.

The presence of family members aged over 65 years is the primary contributor to CHE inequality. This pro-poor contribution indicates that low-income elderly households are more likely to experience CHE. Previous studies showed that the presence of family members aged over 65 years of increases OOP health expenditures, as this category of the population is vulnerable to diseases and health dysfunctions [55]. Although the current reimbursement rate for medical insurance for the elderly is continuously increasing, the costs of nursing care, transportation, and nutrition due to illnesses are not covered by medical reimbursement. Moreover, the problem of aging in China has become severe. Older people (aged 60 or older) are expected to outnumber people between 0 and 14 years of age by 2020 [56]. In addition, the People’s Republic of China’s one-child policy increases the pressure on home care for the elderly. Zhang et al. found that the current medical insurance does not play a significant role in reducing inequality among patients who need long-term care in China [57]. Therefore, reform of the medical insurance system, in addition to integrating the existing medical insurance system, should also consider introducing a medical insurance system for the elderly and covering long-term care services.

Furthermore, hospitalization of a family member is more likely to occur in wealthier households. This phenomenon reduces inequality in CHE, disfavoring the rich. In other words, as poor people use lesser inpatient care, they are less affected by the catastrophic impact of spending on such services. As the use of inpatient services is concentrated in wealthier families, this phenomenon increases the chance of CHE in such families, thus reducing inequality in the number of families facing CHE in different socio-economic groups. Although hospitalization reduces the inequality of CHE occurrences, it is also positively associated with CHE occurrences. Deng et al. found that differentiation in copayment design can influence patients’ medical-care behavior in the Chinese tiered health care system [58]. In the future reform process, the Chinese government should focus on the combination of a tiered health care system and a medical insurance integrated system to reduce unnecessary health expenditures of patients and ultimately reduce the H_cat_.

This study suffered several limitations; hence, the results should be interpreted with great caution. First, the data used for analysis reflect the initial results of the integration policy, but implementation of the integration process requires long-term observation and evaluation. Second, considering the self-selection issue, which may influence actual estimates of the expenditure for CHE. In the future, we will use Heckman’s two-stage model to correct the sample selection bias. Meanwhile, we plan to keep collecting relevant data from the areas of medical insurance integration and compare new data with the results of this study to further analyze the implementation effect of China’s medical insurance integration policy. Third, survey weights were not considered in this study, and the results were based on an unweighted analysis, the odds ratio of which might be smaller than that of considering weight [59]. Finally, clusters were not adjusted in this study, which can lead to underestimation of standard errors [60]. Therefore, one important suggestion is that multi-level studies should be conducted in the future.

## Conclusions

As the medical insurance integration policy is still at the exploratory stage, its effect has limited significance. However, the positive impact of the medical insurance integration system on low-income residents is confirmed. In both IAs or NIAs, CHE shows pro-poor inequality. Medical insurance, urban-rural disparities, growing number of the elderly, and use of health services are associated with inequality in the H_cat_. In the implementation of future insurance integration policies, the Chinese government should focus on the utilization of health services and the gap between urban and rural areas, establishing a reasonable fundraising and payment mechanism. This study’s results also suggest that the Chinese government should consider introducing a medical insurance system for the elderly and covering long-term care services.

## Supplementary information

**Additional file 1: Table S1.** Description of the integrated and unintegrated areas involved in the study.

## Data Availability

Please contact corresponding author for data requests.

## Abbreviations

CHE: Catastrophic health expenditure
CI: Concentration index
CTP: Capacity to pay
G_cat_: The mean gap of catastrophic health expenditure
H_cat_: Catastrophic health expenditure incidence
IA: Integrated area
MPG_cat_: The mean positive gap of catastrophic health expenditure
NHSS: National Health Services Survey
NIA: Non-integrated area
NRCMS: New rural cooperative medical system
OOP: Out-of-Pocket health payments
UEBMI: Urban employee basic medical insurance
URBMI: Urban residents’ basic medical insurance
URRBMI: Urban-rural resident basic medical insurance
USBMI: Universal social basic medical
W^E^_cat_: The weighted H_cat_
WHO: World Health Organization
