# Supplementary material for: Catastrophic health expenditure incidence and its equity in China: a study on the initial implementation of the medical insurance integration system
Source: BMC Public Health. 2019 Dec 30;19:1761. doi: 10.1186/s12889-019-8121-2 (PMC6937839; doi:10.1186/s12889-019-8121-2)
Supplement: Supplementary file 1 — Additional file 1: Table S1. Description of the integrated and unintegrated areas involved in the study. [file 12889_2019_8121_MOESM1_ESM.docx]

**Additional file 1: Description of the integrated and unintegrated areas involved in the study**

Table S1: Description of the integrated and unintegrated areas involved in the study

| Integrated areas | | | | | | | Non-integrated area | | |
| --- | --- | --- | --- | --- | --- | --- | --- | --- | --- |
|  | USBMI | | | URRBMI | | |  |  | |
| Province/ Municipality/ Autonomous Region (ID) | Sample areas | Sample areas ID | Year | Sample areas | Sample areas ID | Year | Province/ Municipality/ Autonomous Region (ID) | Sample areas | Sample areas ID |
| Tianjin (12) |  |  |  | Hebei District | 120105 | 2010 | Beijing City (11) ^‡^ | Dongcheng District | 110101 |
|  |  |  |  | Binhai New Area | 120116 |  |  | Miyun Country | 110228 |
|  |  |  |  | Ji County | 120225 |  |  |  |  |
| Zhejiang (33) |  |  |  | Shangcheng District | 330102 | 2010 | Zhejiang (33) ^†^ | Haishu District | 330203 |
|  |  |  |  | Tonglu County | 330122 | 2010 |  | Huangyan District | 331003 |
|  |  |  |  | Tongxiang City | 330483 | 2003 |  |  |  |
|  |  |  |  | Shengzhou City | 330683 | 2013 |  |  |  |
| Jiangsu (32) |  |  |  | Xishan District | 320205 | 2011 | Jiangsu (32) ^†^ | Pizhou City | 320382 |
|  |  |  |  | Gusu District | 320508 | 2012 |  | Qishuyan District | 320405 |
|  |  |  |  | Yangzhong City | 321182 | 2010 |  | Jinhu County | 320831 |
| Fujian (35) |  |  |  | Jianyang City | 350784 | 2011 | Fujian (35) ^†^ | Yongtai County | 350125 |
|  |  |  |  |  |  |  |  | Ninghua County | 350424 |
|  |  |  |  |  |  |  |  | Yongding County | 350822 |
| Guangdong (44) | Nanshan District | 440305 | 2008 |  |  |  | Guangdong (44) ^†^ | Liwan District | 440103 |
|  |  |  |  | Shunde District | 440606 | 2004 |  | Meijiang District | 441402 |
|  |  |  |  | Sihui City | 441284 | 2012 |  |  |  |
|  |  |  |  | Yangdong County | 441723 | 2011 |  |  |  |
|  |  |  |  | Yingde City | 441881 | 2011 |  |  |  |
|  | Zhongshan city | 442000 | 2010 |  |  |  |  |  |  |
| Hunan (43) |  |  |  | Tianxin District | 430103 | 2011 | Hunan (43) ^†^ | Zhengxiang District | 430408 |
|  |  |  |  |  |  |  |  | Yunxi District | 430603 |
|  |  |  |  |  |  |  |  | Anxiang County | 430721 |
|  |  |  |  |  |  |  |  | Cili County | 430821 |
|  |  |  |  |  |  |  |  | Anren County | 431028 |
|  |  |  |  |  |  |  |  | Hecheng District | 431202 |
|  |  |  |  |  |  |  |  | Yongshun County | 433127 |
| Hubei (42) |  |  |  | Echeng District | 420704 | 2009 | Hubei (42) ^†^ | Qingshan District | 420107 |
|  |  |  |  |  |  |  |  | Xisaishan District | 420203 |
|  |  |  |  |  |  |  |  | Zhuxi County | 420324 |
|  |  |  |  |  |  |  |  | Xiling District | 420502 |
|  |  |  |  |  |  |  |  | Laohekou City | 420682 |
|  |  |  |  |  |  |  |  | Macheng City | 421181 |
|  |  |  |  |  |  |  |  | Hefeng County | 422828 |
| Anhui (34) |  |  |  | Lujiang County | 340124 | 2012 | Anhui (34) ^†^ | Luyang District | 340103 |
|  |  |  |  | Fanchang County | 340222 | 2008 |  | Guzhen County | 340323 |
|  |  |  |  |  |  |  |  | Bagongshan District | 340405 |
|  |  |  |  |  |  |  |  | Daguan District | 340803 |
|  |  |  |  |  |  |  |  | Huangshan District | 341003 |
|  |  |  |  |  |  |  |  | Mengcheng County | 341622 |
| Chongqing (50) |  |  |  | Qianjiang District | 500114 | 2010 | Shaanxi (61) ^‡^ | Lintong District | 610115 |
|  |  |  |  | Zhong Country | 500233 | 2010 |  | Jintai District | 610303 |
|  |  |  |  | Wanzhou District | 500101 | 2010 |  | Mei County | 610326 |
|  |  |  |  | Yuzhong District | 500103 | 2010 |  | Weicheng | 610404 |
|  |  |  |  | shapingba district | 500106 | 2010 |  | Hanyin County | 610921 |
| Sichuan (51) |  |  |  | Qingyang District | 510105 | 2009 | Sichuan (51) ^†^ | Daan District | 510304 |
|  |  |  |  |  |  |  |  | Jiangyang District | 510502 |
|  |  |  |  |  |  |  |  | Yanting County | 510723 |
|  |  |  |  |  |  |  |  | Anju District | 510904 |
|  |  |  |  |  |  |  |  | Langzhong City | 511381 |
|  |  |  |  |  |  |  |  | Dongpo District | 511402 |
| Ningxia (64) |  |  |  | Xixia District | 640105 | 2012 | Hebei (13) ^‡^ | Lubei District | 130203 |
|  |  |  |  | Longde County | 640423 | 2011 |  | Wuan City | 130481 |
|  |  |  |  | Zhongning County | 640521 | 2012 |  | Tang County | 130627 |
|  |  |  |  |  |  |  |  | Fengning Manchu Autonomous County | 130826 |
| Inner Mongolia (15) |  |  |  | Jungar Banner | 150622 | 2012 | Inner Mongolia ^†^ (15) | Kalaqin Banner | 150428 |
|  |  |  |  |  |  |  |  | Kailu Country | 150523 |
| Xinjiang (65) |  |  |  | Baijiantan District | 650204 | 2009 | Xinjiang (65) ^†^ | Tianshan District | 650102 |
|  |  |  |  |  |  |  |  | Saybagh District | 650103 |
|  |  |  |  |  |  |  |  | Hotan County | 653221 |
|  |  |  |  |  |  |  |  | Xinyuan County | 654025 |

Note: USBMI: universal social basic medical insurance; URRBMI: urban-rural resident basic medical insurance; Year: the time of implementation of the medical insurance integration system; † non-integration pilots of the province/ municipality/ autonomous region where the integrated areas are located; ‡ comparable regions by per capita GDP (as of 2012) are used for the analysis.
